# Cortical morphological networks for profiling autism spectrum disorder using tensor component analysis

**DOI:** 10.3389/fneur.2024.1391950

**Published:** 2024-07-04

**Authors:** Kubra Cengiz, Islem Rekik

**Affiliations:** ^1^Faculty of Computer and Informatics, Istanbul Technical University, İstanbul, Türkiye; ^2^BASIRA Lab, Imperial-X and Department of Computing, Imperial College London, London, United Kingdom

**Keywords:** cortical morphological networks, tensor component analysis, brain connectivity, autism spectrum disorder, multi-view profiling

## Abstract

Atypical neurodevelopmental disorders such as Autism Spectrum Disorder (ASD) can alter the cortex morphology at different levels: (i) a low-order level where cortical regions are examined individually, (ii) a high-order level where the relationship between two cortical regions is considered, and (iii) a multi-view high-order level where the relationship between regions is examined across multiple brain views. In this study, we propose to use the emerging multi-view cortical morphological network (CMN), which is derived from T1-w magnetic resonance imaging (MRI), to profile autistic and typical brains and pursue new ways of fingerprinting ‘cortical morphology' at the intersection of ‘network neuroscience'. Each CMN view models the pairwise morphological dissimilarity at the connection level using a specific cortical attribute (e.g., thickness). Specifically, we set out to identify the inherently most representative morphological connectivities shared across different views of the cortex in both autistic and normal control (NC) populations using tensor component analysis. We thus discover the connectional profiles of both populations shared across different CMNs of the left and right hemispheres, respectively. One of the most representative morphological cortical attributes for assessing the abnormal brain structures in patients with ASD is cortical thickness. The most representative morphological connectivities in multi-view CMN population of normal control and ASD subjects, respectively, and in both left and right hemispheres within the temporal, frontal, and insular lobes of individuals with ASD. These representative connectivities are corresponded to specific clinical features observed in individuals with ASD.

## 1 Introduction

Cortical morphological networks (CMNs), emerging at the intersection of “network neuroscience” and “cortical morphology”, are networks which portray dissimilarity among network nodes in morphology, and they have been demonstrating to have utility in diagnosing neurological disorders (1, 2), estimating network atlases of the cortex (3, 4), and investigating gender differences in cortical morphology (5). In contrast with *conventional* functional networks derived from functional magnetic resonance imaging (fMRI) and structural networks derived from diffusion-weighted MRI (6), cortical morphological networks provide new network-based representations of the cerebral cortex, which are derived from *solely* baseline T1-weighted MRI based on different cortical attributes (e.g., cortical thickness and sulcal depth). Indeed, previous studies showed that brain morphology can be affected by different psychiatric disorders, in particular the cortex. Although complex and variable, the morphology of the cortical gyri and sulci at birth predicted pathological functioning in certain developmental and neuropsychiatric disorders (7), thereby highlighting that brain morphology and function are intertwined. Notably, changes in function can elicit changes in morphology and structure and vice versa. Indeed, according to the tension theory of cerebral cortex morphogenesis, network changes in the morphological attributes of the brain (e.g., cortical surface attributes such as curvature) reflect the underlying changes in the structural and functional connectivity (8) and can be studied without the need for costly and time-consuming imaging of patients using advanced fMRI and dMRI facilities.

**Figure 1:**
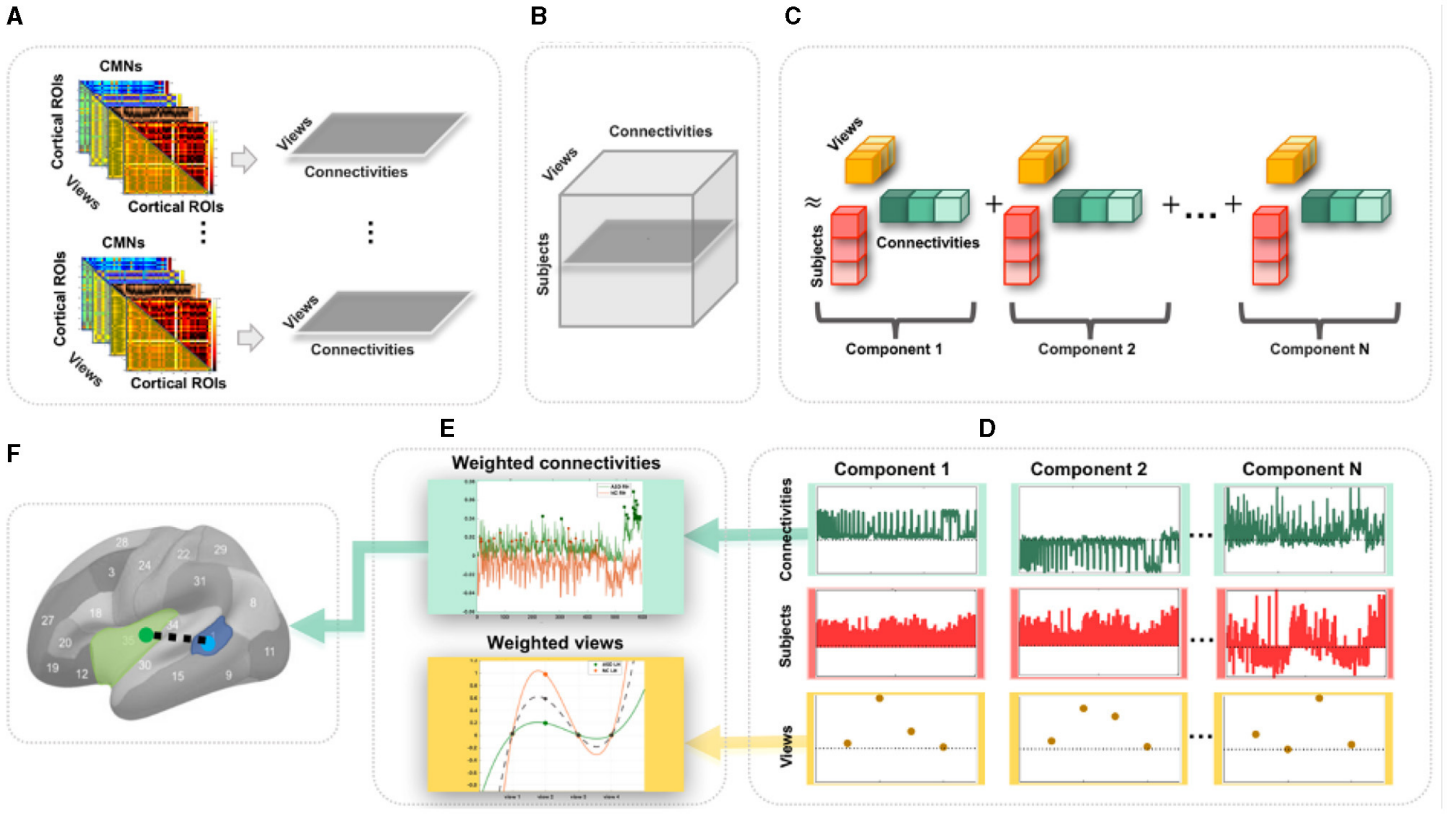
Proposed pipeline to discover the most representative connections for profiling a population of multi-view cortical morphological networks (CMNs). **(A)** For each subject, we define four cortical morphological networks (views) encoding the dissimilarity in morphology between pairs of cortical regions. Next, we generate view-specific vector by vectorizing the off-diagonal lower triangular matrix of each CMN. Hence, for each subject, multi-view CMN (gray horizontal plane) combines view-specific vectors using specific cortical attributes. **(B)** Construction of a third-order population-based tensor, where each horizontal plane (dark gray) represents a multi-view CMN (views × connectivities) of a single subject. **(C)** Tensor component analysis (TCA). The tensor is approximated by a sum of outer products of three rank-one vectors, producing an additional set of low-dimensional factors (subject, view, and connectivity factors). **(D)** Extraction of the average subject (red), view (yellow), and connectivity (green) factors for different TCA components across five random partitions of the data for reproducibility. **(E)** Display of the weights (magnitudes) of factors. These low-dimensional view-based and connectivity-based factors' weights are used for profiling healthy and autistic cortices. **(F)** Discovery of the most representative connectivity shared across morphological views for a given CMN population. Here, we display the most representative connectivity in profiling the autistic left hemisphere.

Undoubtedly, traditional functional and structural network neuroscience (6) has substantially advanced our understanding of neurological disorders that involve atypical changes in brain connectivity. In particular, autism spectrum disorder (ASD) has been widely investigated using resting-state fMRI (9) and dMRI (10), offering insights into its biological mechanisms. Nevertheless, ASD remains a behaviorally defined syndrome with no reliable biological markers (11). However, we note that different cortical attributes identified cortical shape-related alterations manifesting during ASD disorder progression such as temporal and parietal cortical thinning (12). As such, the emerging multi-view CMNs, where each CMN is derived from a *specific cortical attribute* (e.g., thickness) producing a specific *network view*, may eventually provide complementary insights into the etiology of the autistic cortex. Although the neuroscience network literature provides preliminary evidence for substantiating and fingerprinting the autistic brain connectivity (9, 10), these were limited to investigating functional and structural networks (at the connectivity level) and overlooked the cross-network interaction (at the network level). To address these limitations, we propose to use *multi-view CMNs* to discover the connectional profiles of the autistic and healthy cortices, respectively, that are shared across four cortical attributes (i.e., maximum principal curvature, cortical thickness, sulcal depth network, and average curvature). Each subject is hence represented by a set of cortical morphological networks, each encoded in a connectivity matrix (Figure 1A). Next, for each subject, we extract the connectivity values in each CMN off-diagonal lower triangular matrix, thereby defining a view-specific vector. Drawing on the wealth of machine learning approaches for multi-view data analysis, we root our population-based multi-view CMN analysis framework in the robust mathematical theory of tensor component analysis (TCA) of multi-view datasets (13). Specifically, we encode a population of CMNs in a three-dimensional *tensor structure*, where the first dimension represents subjects, the second dimension denotes morphological connectivities, and the third dimension defines the network view. It is a third-order tensor (three-dimensional array) in which each entry indicates the morphological connectivity of a particular subject with a particular view on a particular pair of cortical region of interest (ROI) (Figures 1A, B). This nicely captures the connectivity structure and multi-view network complementarity across subjects and avoids the loss of information using concatenation, where all CMN views are vectorized and then concatenated in a single long feature vector.

To map or profile high-dimensional biological data, one can leverage data decomposition and dimensionality reduction such as principal component analysis, which map the high-dimensional data onto a new space where a few and most meaningful components (also called dimensions or factors) are estimated and inherent data profiles are charted. Since biological networks are non-linear and have non-orthogonal properties (14), morphological connectivities might overlap between cortical regions and could be correlated, yielding a non-orthogonal structure that cannot be recovered by conventional mapping techniques such as PCA or independent component analysis (ICA) (13). In addition, while TCA is a simple generalization of PCA, its theoretical properties are strikingly more favorable in comparison to both PCA and ICA which are fundamentally matrix decomposition methods. Consequently, we leverage TCA to profile ASD and NC multi-view brain networks more effectively and circumvent the need to average the cortex tensor across views. Indeed, TCA is able to achieve a simultaneous and shared dimensionality reduction across subjects, connectivities, and views, reducing *N*_*s*_ (subjects) × *N*_*c*_ (connectivities) × *N*_*v*_ (views) to *N*_*t*_ (tensor components) × (*N*_*s*_+*N*_*c*_+*N*_*v*_) (13) while capturing view-to-view connectional variability (Figure 1C). In this study, we leverage TCA to provide unprecedented profiling of ASD and NC based on multi-view connectional brain maps in an unsupervised, fully data-driven fashion. Our goal was to identify the most representative morphological connectivities shared across different views of the CMNs of the left and right hemispheres in both autistic and normal control (NC) populations using the TCA method in an unsupervised, fully data-driven fashion. The method was applied to a large population sample of brain imaging data, the Autism Imaging Data Exchange I (ABIDE I).

## 2 Methods

### 2.1 Dataset

We conducted a thorough examination of brain imaging data from ASD patients, which was taken from the global multi-site database known as Autism Brain Imaging Data Exchange (ABIDE I), comprising 341 subjects of which 155 (15 female(F), 140 male(M)) with 16.92 ± 6.38 age ASD, and 186 (31 F, 155 M) with 16.65 ± 6.06 age NC subjects (15) (*N*_*s*_ = 155 for ASD and 186 for NC). Each subject has structural T1-w MRI. We used FreeSurfer (16) to extract both right and left hemispheres (RH and LH) for each subject and then parcellate each into *N*_*r*_ = 35 cortical regions of interest using Desikan-Killiany Atlas (17). We derive *N*_*v*_ = 4 CMNs from specific cortical measurements for each subject as introduced in the study by (1): (1) maximum principal curvature, (2) cortical thickness network, (3) sulcal depth network, and (4) average curvature network. Next, for each cortical attribute and for each ROI, we compute an average cortical attribute. To define the morphological connection between two ROIs, we compute the absolute distance between average cortical measurement in both ROIs. In a CMN, when two ROIs *R*_*i*_ and *R*_*j*_, become more similar in morphology, their morphological connectivity nears zero (Supplementary Table 1, Supplementary Data). By vectorizing the off-diagonal upper triangular part of each CMN, we generate a connectivity vector of size *N*_*c*_ = *N*_*r*_×(*N*_*r*_−1)/2 (i.e., 595 connectivities for *N*_*r*_ = 35). We would like to note that the method and experimental protocols were carried out using the public Autism Brain Imaging Data Exchange (ABIDE) dataset. Informed consent was obtained from all ABIDE subjects or, if subjects are younger than 18 years, from a parent and/or legal guardian.

### 2.2 CMN profiling using TCA

**Figure 2:**
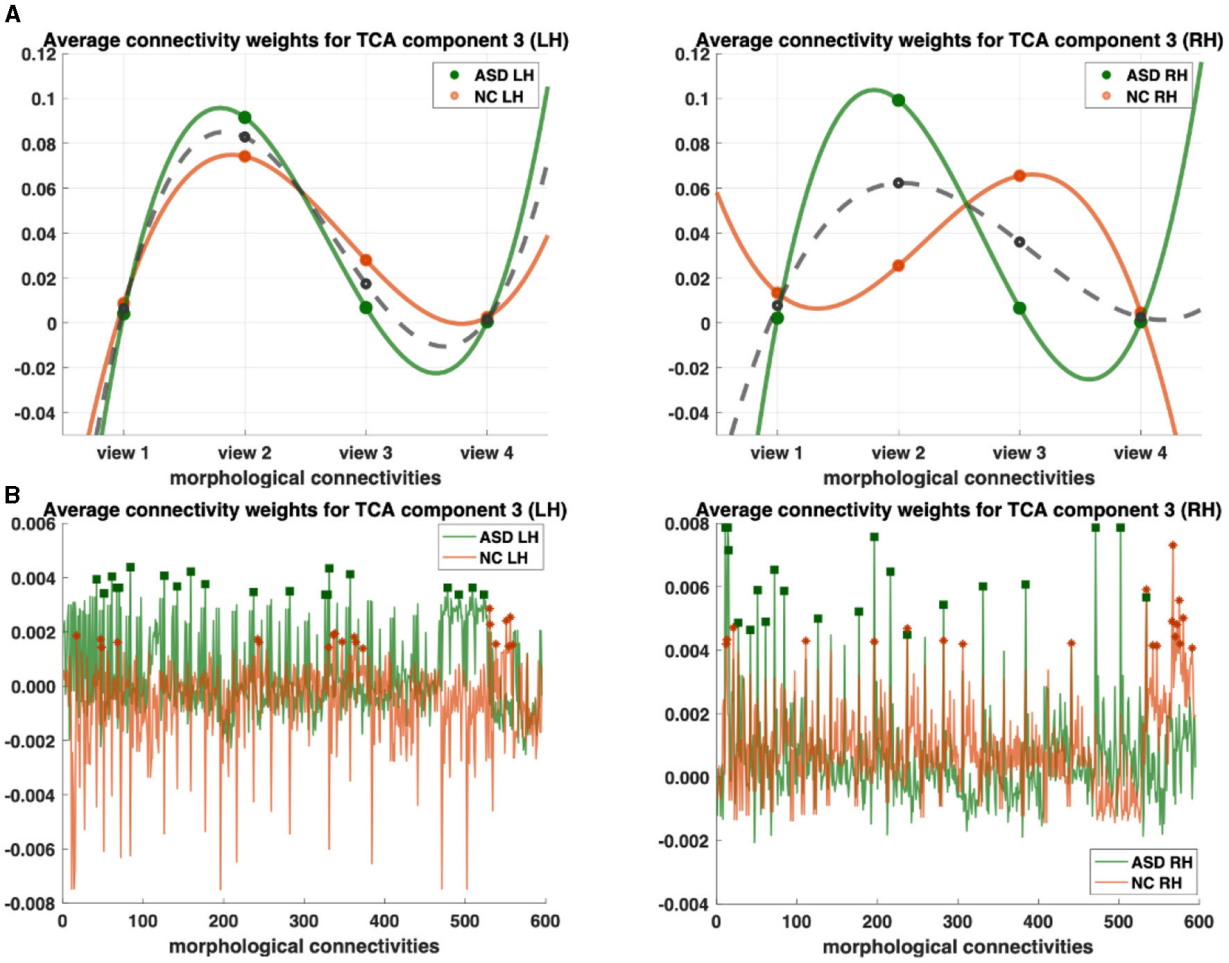
Profiling of autism spectrum disorder (ASD) and normal control (NC) populations and discovery of most representative connections shared across multi-view cortical morphological networks (CMNs). **(A)** Visualization of the view factors by tensor component analysis (TCA) of the multi-view CMN population of healthy and autistic subjects, respectively, and in both the left and right hemispheres (LH and RH). We display the view factors using TCA component 3. Each line (e.g., solid green for ASD) profiles the relevance of each view in representing the given multi-view CMN population. The dashed gray line is the average of ASD and NC green and orange lines. **(B)** Visualization of the view factors by tensor component analysis (TCA) of the multi-view CMN population of normal control and ASD subjects, respectively, and in both the left and right hemispheres (LH and RH). We display the view factors using TCA component 3. Each line (e.g., solid green for ASD) profiles the relevance of each view in representing the given multi-view CMN population. Discovery of the most representative cortical connectivity in profiling ASD and NC populations. Filled rectangles and stars display the top 20 most representative connectivities for ASD and NC populations, respectively.

**Figure 3:**
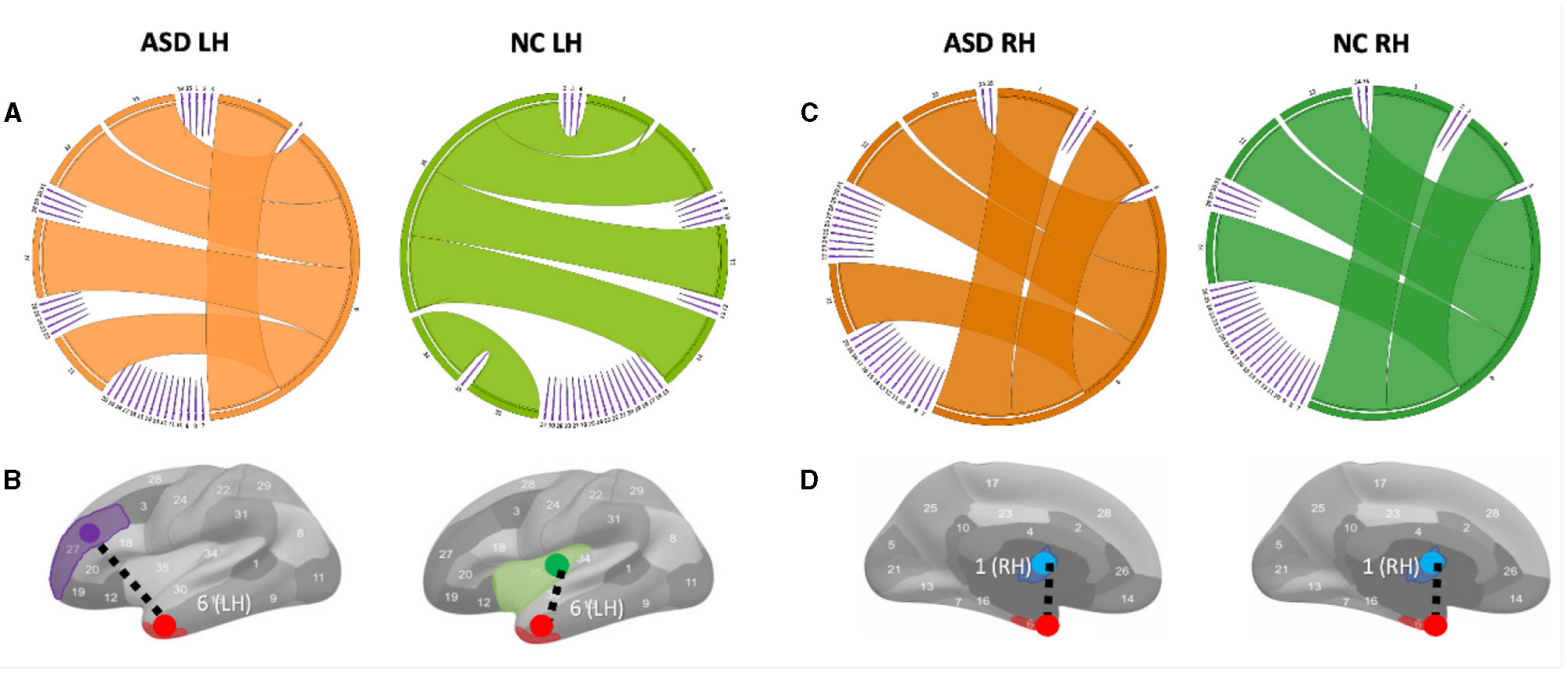
Circular graphs of the top five most representative morphological connections in profiling ASD and NC populations using TCA third component for view 2. **(A)** The edge thickness quantifies the representativeness of each connection (the higher the thickness, the more important the spotted connection is in representing the morphological brain connectome). Identifying the most representative morphological cortical connection in profiling ASD LH and NC LH (left to right) populations. **(B)** The most representative brain connection for ASD cortices is (entorhinal cortex (EC) (red) ↔ rostal middle frontal gyrus (purple)) (LH). The most representative brain connections for NC cortices is (EC↔ insula cortex (green)) (LH). All results in this figure present the average results across 50 random partitions of each population to increase robustness against data perturbation. **(C)** Circular graphs of the top five most representative morphological connections in profiling ASD RH and NC RH (left to right) populations using TCA third component for view 2. **(D)** The most representative brain connection for ASD cortices is (bank of the superior temporal sulcus (STS) (blue) ↔ EC) (RH). The most representative brain connections for NC cortices is (STS↔EC)(LH).

We map each population-based high-dimensional CMN tensor of dimension *N*_*s*_×*N*_*c*_×*N*_*v*_ into a low-dimensional space with *N*_*t*_ = 4 dimensions (components). Indeed, TCA approximates the CMN tensor as a sum of outer products of three vectors, producing an additional set of low-dimensional factors (subject factors, connectivity factors, and view factors) that capture how brain connectivity changes across views (13) (Figure 1C). Specifically, we fit a tensor decomposition model (13) to identify a set of low-dimensional components describing variability along each of these three axes. By allowing a multi-dimensional space of possible connectivities to different view factors, TCA can capture a rich diversity of changing multi-brain connection patterns across views for each hemisphere in healthy and disordered cortices (Figure 1D). We visualized the connectivity and view factor using TCA to profile unrivaled structure of populations (Figure 1E), as shown in Figures 2A, B. We discover that shared morphological connectivity between healty and austistic population and brain graph represents the morphological connections between ROIs (Figure 1F), as shown in Figure 3. We randomly split available subjects in each population into five random sets. For rigorous TCA reproducibility, we report the *average* profiles discovered across five random data partitions. This allows to avoid population-driven bias by perturbing the set of the subjects to learn from TCA model and derive our analyses.

## 3 Results

We profile ASD/NC at two fronts: (1) individually across view and connectivity factors, respectively, to learn their corresponding shared patterns across independent subjects and (2) jointly across all subject, view, and connectivity factors. First, we display the view factors of the third components for ASD (light orange LH/dark orange RH) and NC (light green LH/ dark green RH) for each hemisphere (Figure 2). For each CMN view, we display its average weight of view factors for ASD and NC (dashed gray), respectively (Figure 2A). In terms of representativeness, we note that view 2 (i.e., cortical thickness network) has the highest weight for each population across components. For example, in the left hemisphere, the highest weights of view factors are of views 2 and 3, which were captured by components 3 and 1 (*p*−*value* < 0.05 using two-tailed paired t-test), respectively (Figure 3). In the right hemisphere, view 2 was found as most representative across views using components 3 and 4 (*p*−*value* < 0.05 using two-tailed paired t-test). In terms of discriminative profiling of ASD and NC populations, both view 3 (i.e., sulcal depth) for LH components 1 and 3 and view 2 for RH component 3 achieved the largest margins between factors' weights, indicating that both populations can be easily separated by these views even though the tensor decomposition has no knowledge of the population labels i.e., this is discovered in a fully data-driven unsupervised manner. The weights of connectivity factors for ASD (green) and NC (orange) were displayed for the component 3 and in both hemispheres (Figure 3). After sorting the connectivity factor weights in descending order, we select the 20 largest weights which are highlighted using filled rectangles (ASD) and stars (NC) in Figure 2B. In terms of discriminative profiling of ASD and NC populations, for each hemisphere, component 3 displayed the largest margins between the largest weights of connectivity factors, indicating that both populations can be linearly separated by these components (i.e., we can also easily fit a line to discriminate them) (Supplementary Figures 7–9).

## 4 Discussion

Variations in shared morphological connectivity weights can be represented in a graph by the thickness of each edge. We display two graph representations: circular and brain cortical hemisphere (Figure 3). We visualize the top five most representative morphological connectivities profiling ASD and NC populations for the left hemisphere (Figure 3A) and right hemisphere (Figure 3C). The thickness of each cortical connection reflects its importance in mapping (or non-linearly representing) other cortical connections across the four cortical views. The most representative high-order morphological connections for ASD left hemisphere include (1) (entorhinal cortex (EC)↔rostral middle frontal gyrus), (2) (unmeasured corpus callosum (CC)↔EC), (3) (EC↔frontal pole (FP)), (4) (EC↔temporal pole (TP)), and (5) (EC↔pericalcarine cortex (PC)), as shown in Figure 3A. For the right hemisphere, the top most representative cortical connections include (1) (STS↔EC), (2) (CC↔EC), (3) (EC↔FP), (4) (EC↔TP), and (5) (EC↔PC), as shown in Figure 3C. These results were statistically significant with a *p*−*value* < 0.05 using two-tailed paired t-test on ASD and NC groups (Supplementary Figures 4, 5).

To the best of our knowledge, we found no difference in the most representative cortical connection profiling NC RH and ASD RH populations, which connected the STS and the temporal pole (Figures 3C, D). However, for the ASD RH population, (STS↔EC) was identified as the most representative connection, which might be related to the correlation of the EC with the severity of symptomatology of ASD (18). A meta-analysis (19) showed that in ASD, there are volume reductions in the hippocampal area, which includes the entorhinal cortex found in the medial regions of the temporal lobe. This entorhinal cortex is linked with several other cortices and is the main facilitator for the transfer of cortical information in and out of the actual hippocampus (20). This area is vital for memory processing, so any reductions in volume could potentially lead to issues with episodic memory (18). The superior temporal gyrus, along with its neighboring structure, and the superior temporal sulcus, play a role in non-linguistic social cognition. This includes recognizing behaviors and responding to social information. Abnormalities, such as cortical thinning, reduced gyrification, and less sulcus depth, can contribute to the challenges in social interaction experienced in ASD (21). Specifically, cortical thinning in the right superior temporal gyrus is linked with higher Social Responsiveness Scale (SRS) scores, pointing to more significant social communication difficulties (22). (CC↔EC) was found as the second most representative morphological connection in profiling ASD RH, which is in line with the study by (23) arguing that CC was mostly affected in brain structures in ASD and CC; area, volume, and white matter (WM) density are lower in autism than in typical development. We also discovered the (EC↔rostral middle frontal gyrus) morphological connection as the most representative in profiling ASD LH Figure 3B. (24) that age-related cortical thinning in ASD subjects increases in the right paracentral cortex and left pars opercularis, rostral middle frontal gyrus, and frontal pole compared with typical subjects. Although our findings gave novel insights into the role of CMNs in profiling the cortex on a connectional level, network neuroscience demands not only to examine the brain using conventional T1-w MRI but it also calls for underpinning holistic profiles of its morphology, structure, and function using multimodal neuroimaging.

## Data availability statement

The original contributions presented in the study are included in the article/Supplementary material, further inquiries can be directed to the corresponding author.

## Ethics statement

Ethical review and approval was not required for the study on human participants in accordance with the local legislation and institutional requirements. Written informed consent from the [patients/ participants OR patients/participants legal guardian/next of kin] was not required to participate in this study in accordance with the national legislation and the institutional requirements.

## Author contributions

KC: Formal analysis, Methodology, Validation, Visualization, Writing – original draft, Writing – review & editing. IR: Conceptualization, Methodology, Resources, Supervision, Writing – review & editing.
