# Supplementary material for: Cortical morphological networks for profiling autism spectrum disorder using tensor component analysis
Source: Front Neurol. 2024 Jul 4;15:1391950. doi: 10.3389/fneur.2024.1391950 (PMC11254826; doi:10.3389/fneur.2024.1391950)
Supplement: Supplementary file 1 [file Table_1.pdf]

Supplementary Material

1 SUPPLEMENTARY DATA

1.1 Morphological Brain Network Definition

Following the cortical surface parcellation into R anatomical regions, for each ROI R_i, we average the cortical attribute a across all vertices v in R_i as follows:

$$\frac{1}{\#\{v \in R_i\}} \sum_{v \in R_i} a(v) \tag{S1}$$

where $\#v \in R_i$ denotes the number of vertices v belonging to ROI R_i, and $a(v)$ the cortical attribute value assigned to vertex v. Ultimately, to define the morphological connection $Na(i,j)$ in network Na between ROIs R_i and R_j, we compute the absolute difference between averaged cortical attributes in both ROIs:

$$N_a(i,j) = \left| \frac{1}{\#\{v \in R_i\}} \sum_{v \in R_i} a(v) - \frac{1}{\#\{v \in R_j\}} \sum_{v \in R_j} a(v) \right| \tag{S2}$$

Given R cortical regions in each hemisphere, the size of each fully connected morphological network is $R \times R$. We note that according to our definition, as two ROIs R_i and R_j become more similar in morphology, $N_a(i,j)$ tends to 0.

2 SUPPLEMENTARY TABLES AND FIGURES

2.1 Tables

	NC	ASD
Male	140	155
Female	15	31
Total	155	186
Mean Age	16.92	16.65
Std Age	6.38	6.06

Table S1. The demographic information for ASD group and NC group.

2.2 Figures

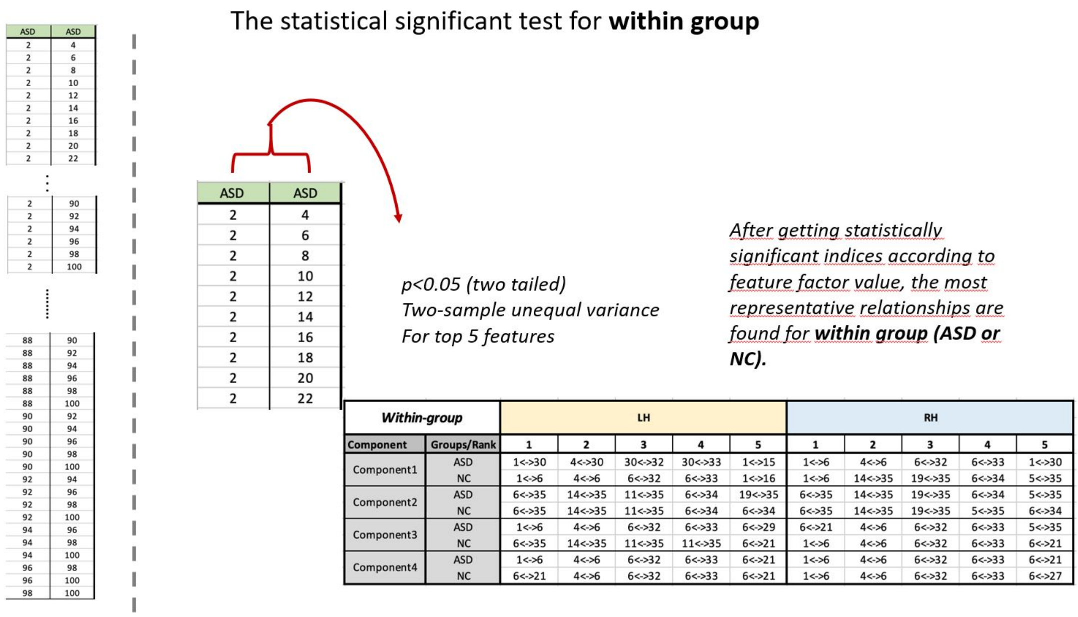

Within-group		LH					RH				
**Component**	**Groups/Rank**	**1**	**2**	**3**	**4**	**5**	**1**	**2**	**3**	**4**	**5**
Component1	ASD	1<->30	4<->30	30<->32	30<->33	1<->15	1<->6	4<->6	6<->32	6<->33	1<->30
	NC	1<->6	4<->6	6<->32	6<->33	1<->16	1<->6	14<->35	19<->35	6<->34	5<->35
Component2	ASD	6<->35	14<->35	11<->35	6<->34	19<->35	6<->35	14<->35	19<->35	6<->34	5<->35
	NC	6<->35	14<->35	11<->35	6<->34	6<->34	6<->35	14<->35	19<->35	5<->35	6<->34
Component3	ASD	1<->6	4<->6	6<->32	6<->33	6<->29	6<->21	4<->6	6<->32	6<->33	5<->35
	NC	6<->35	14<->35	11<->35	11<->35	6<->21	1<->6	4<->6	6<->32	6<->33	6<->21
Component4	ASD	1<->6	4<->6	6<->32	6<->33	6<->21	1<->6	4<->6	6<->32	6<->33	6<->21
	NC	6<->21	4<->6	6<->32	6<->33	6<->21	1<->6	4<->6	6<->32	6<->33	6<->27

Figure S1. The most representative relationships are found for within group (ASD or NC) after getting statistically significant (p<0.05 two tailed unequal variance for top 5 features) indices according to feature factor value.

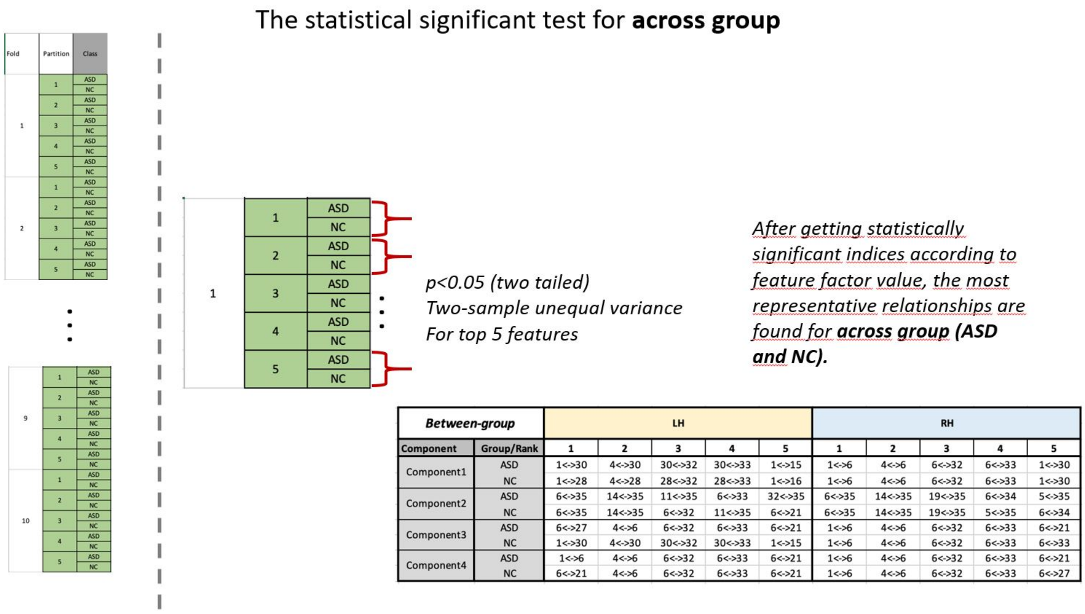

Between-group		LH					RH				
**Component**	**Group/Rank**	**1**	**2**	**3**	**4**	**5**	**1**	**2**	**3**	**4**	**5**
Component1	ASD	1<->30	4<->30	30<->32	30<->33	1<->15	1<->6	4<->6	6<->32	6<->33	1<->30
	NC	1<->28	4<->28	28<->32	28<->33	1<->16	1<->6	4<->6	6<->32	6<->33	1<->30
Component2	ASD	6<->35	14<->35	11<->35	6<->33	32<->35	6<->35	14<->35	19<->35	6<->34	5<->35
	NC	6<->35	14<->35	6<->32	11<->35	6<->21	6<->35	14<->35	19<->35	5<->35	6<->34
Component3	ASD	6<->27	4<->6	6<->32	6<->33	6<->21	1<->6	4<->6	6<->32	6<->33	6<->21
	NC	1<->30	4<->30	30<->32	30<->33	1<->15	1<->6	4<->6	6<->32	6<->33	6<->33
Component4	ASD	1<->6	4<->6	6<->32	6<->33	6<->21	1<->6	4<->6	6<->32	6<->33	6<->21
	NC	6<->21	4<->6	6<->32	6<->33	6<->21	1<->6	4<->6	6<->32	6<->33	6<->27

Figure S2. The most representative relationships are found for across group (ASD and NC) after getting statistically significant (p<0.05 two tailed unequal variance for top 5 features) indices according to feature factor value.

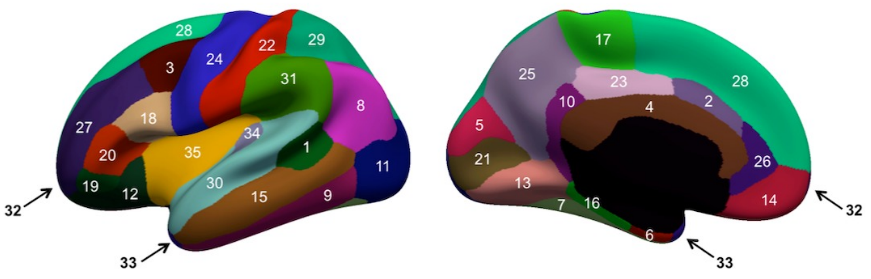

**1.** Bank of the Superior Temporal Sulcus	**2.** Caudal Anterior-cingulate Cortex
**3.** Caudal Middle Frontal Gyrus	**4.** Unmeasured Corpus Callosum
**5.** Cuneus Cortex	**6.** Entorhinal Cortex
**7.** Fusiform Gyrus	**8.** Inferior Parietal Cortex
**9.** Inferior Temporal Gyrus	**10.** Isthmus-cingulate Cortex
**11.** Lateral occipital cortex	**12.** Lateral orbital frontal cortex
**13.** Lingual gyrus	**14.** Medial orbital frontal cortex
**15.** Middle temporal gyrus	**16.** Parahippocampal gyrus
**17.** Paracentral lobule	**18.** Pars opercularis
**19.** Pars orbitalis	**20.** Pars triangularis
**21.** Pericalcarine cortex	**22.** Postcentral gyrus
**23.** Posterior-cingulate cortex	**24.** Precentral gyrus
**25.** Precuneus cortex	**26.** Rostral anterior cingulate cortex
**27.** Rostral middle frontal gyrus	**28.** Superior frontal gyrus
**29.** Superior parietal cortex	**30.** Superior temporal gyrus
**31.** Supramarginal gyrus	**32.** Frontal pole
**33.** Temporal pole	**34.** Transverse temporal cortex
**35.** Insula cortex	

Figure S3. Cortical brain regions of interest used for morphological brain network reconstruction. The numbers with corresponding names can be linked to the tables in (Fig. S1 and Fig. S2).

The mean sulcal depth brain view (view 3)

Between-group		LH					RH				
**Component**	**Group/Rank**	**1**	**2**	**3**	**4**	**5**	**1**	**2**	**3**	**4**	**5**
Component1	ASD	1<->30	4<->30	30<->32	30<->33	1<->15	1<->6	4<->6	6<->32	6<->33	1<->30
	NC	1<->28	4<->28	28<->32	28<->33	1<->16	1<->6	4<->6	6<->32	6<->33	1<->30
Component2	ASD	6<->35	14<->35	11<->35	6<->33	32<->35	6<->35	14<->35	19<->35	6<->34	5<->35
	NC	6<->35	14<->35	6<->32	11<->35	6<->21	6<->35	14<->35	19<->35	5<->35	6<->34
Component3	ASD	6<->27	4<->6	6<->32	6<->33	6<->21	1<->6	4<->6	6<->32	6<->33	6<->21
	NC	1<->30	4<->30	30<->32	30<->33	1<->15	1<->6	4<->6	6<->32	6<->33	6<->33
Component4	ASD	1<->6	4<->6	6<->32	6<->33	6<->21	1<->6	4<->6	6<->32	6<->33	6<->21
	NC	6<->21	4<->6	6<->32	6<->33	6<->21	1<->6	4<->6	6<->32	6<->33	6<->27

Within-group		LH					RH				
**Component**	**Groups/Rank**	**1**	**2**	**3**	**4**	**5**	**1**	**2**	**3**	**4**	**5**
Component1	ASD	1<->30	4<->30	30<->32	30<->33	1<->15	1<->6	4<->6	6<->32	6<->33	1<->30
	NC	1<->6	4<->6	6<->32	6<->33	1<->16	1<->6	14<->35	19<->35	6<->34	5<->35
Component2	ASD	6<->35	14<->35	11<->35	6<->34	19<->35	6<->35	14<->35	19<->35	6<->34	5<->35
	NC	6<->35	14<->35	11<->35	6<->34	6<->34	6<->35	14<->35	19<->35	5<->35	6<->34
Component3	ASD	1<->6	4<->6	6<->32	6<->33	6<->29	6<->21	4<->6	6<->32	6<->33	5<->35
	NC	6<->35	14<->35	11<->35	11<->35	6<->21	1<->6	4<->6	6<->32	6<->33	6<->21
Component4	ASD	1<->6	4<->6	6<->32	6<->33	6<->21	1<->6	4<->6	6<->32	6<->33	6<->21
	NC	6<->21	4<->6	6<->32	6<->33	6<->21	1<->6	4<->6	6<->32	6<->33	6<->27

Figure S4. The most representative relationships are found for across group (ASD and NC) after getting statistically significant (p<0.05 two tailed unequal variance for top 5 features) components for view 3.

The most representative brain connection for view 3 (the mean sulcal depth brain view**)**

Across groups	ASD LH	NC LH	ASD RH	NC RH
	6<->27	1<->30	1<->6	1<->6
	Entorhinal Cortex<->Rostral middle frontal gyrus	Bank of the Superior Temporal Sulcus<->Superior temporal gyrus	Bank of the Superior Temporal Sulcus<->Entorhinal Cortex	Bank of the Superior Temporal Sulcus<->Entorhinal Cortex
**Within Groups**	**ASD LH**	**NC LH**	**ASD RH**	**NC RH**
	1<->6	6<->35	6<->21	1<->6
	Bank of the Superior Temporal Sulcus<->Entorhinal Cortex	Entorhinal Cortex<->Insula cortex	Entorhinal Cortex<->Pericalcarine cortex	Bank of the Superior Temporal Sulcus<->Entorhinal Cortex

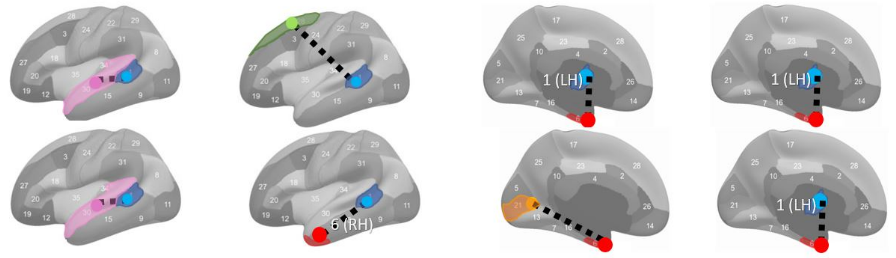

Figure S5. The most representative brain connection across group (ASD and NC) and within group (ASD or NC) for both hemisphere after getting statistically significant (p<0.05 two tailed unequal variance for top 5 features) components for view 3.

The most representative brain connection for view 3 (the mean sulcal depth brain view)

Across groups	ASD LH	NC LH	ASD RH	NC RH
	6<->27	1<->30	1<->6	1<->6
	Entorhinal Cortex<->Rostral middle frontal gyrus	Bank of the Superior Temporal Sulcus<->Superior temporal gyrus	Bank of the Superior Temporal Sulcus<->Entorhinal Cortex	Bank of the Superior Temporal Sulcus<->Entorhinal Cortex
**Within Groups**	**ASD LH**	**NC LH**	**ASD RH**	**NC RH**
	1<->6	6<->35	6<->21	1<->6
	Bank of the Superior Temporal Sulcus<->Entorhinal Cortex	Entorhinal Cortex<->Insula cortex	Entorhinal Cortex<->Pericalcarine cortex	Bank of the Superior Temporal Sulcus<->Entorhinal Cortex

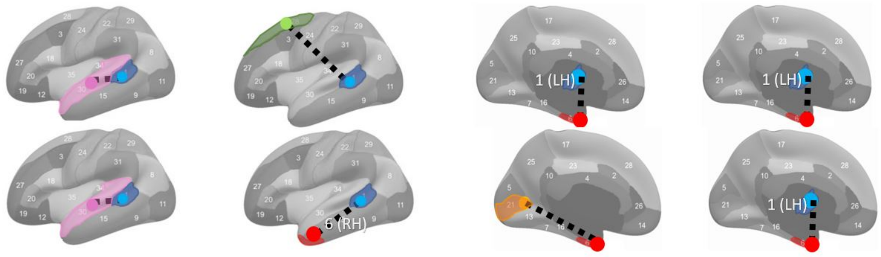

Figure S6. The most representative brain connection across group (ASD and NC) and within group (ASD or NC) for both hemisphere after getting statistically significant (p<0.05 two tailed unequal variance for top 5 features) components for view 2.
